# Miss R – Testosterone Secreting Tumour, Gender Incongruence or Munchausen’s Syndrome? A Case Report

**DOI:** 10.1192/bjo.2025.10749

**Published:** 2025-06-20

**Authors:** Hannah Pasha Memon, Sumerah Jabeen

**Affiliations:** 1 North East London NHS Foundation Trust, London, United Kingdom; 2 Patel Hospital, Karachi, Pakistan

## Abstract

Aims:

Gender variance is a prevalent yet complex phenomenon that continues to generate significant academic and clinical interest. Historically, the diagnosis of Gender Identity Disorder was classified under Mental and Behavioural Disorders in the International Classification of Diseases (ICD). This has since been revised to 'Gender Incongruence’ and relocated to the Conditions Related to Sexual Health section in ICD–11. Despite these advancements, the psychopathology of individuals experiencing distress related to gender incongruence remains insufficiently understood. Countries like Pakistan where sex reassignment procedures are illegal, provide a unique lens through which to explore the psychological experiences of individuals with gender incongruence.

**Methods:** This report details the case of a 20-year-old woman who presented with a testosterone-secreting tumour. Previously identifying and living as a heterosexual female, she reported experiencing homosexual encounters, emotional distress, confusion regarding her gender identity, and an increasing openness to adopting a male identity. Despite surgical interventions, her testosterone levels initially remained elevated and later surged, leading to a diagnosis of Munchausen’s Syndrome (Factitious Disorder).

**Results:** Munchausen’s Syndrome/Factitious Disorder: Munchausen’s syndrome involves intentional fabrication or induction of illness without external rewards, distinguishing it from malingering. Features include inconsistencies in patient history, overdramatic presentations, and willingness to undergo invasive procedures. While Ms R’s case lacked some typical features, her persistence in seeking medical attention and willingness to undergo surgical procedures supported the diagnosis.

Gender Incongruence: ICD–11 defines Gender Incongruence as persistent mismatch between experienced gender and assigned sex, often prompting medical interventions. In Pakistan, gender incongruence is stigmatized, compounded by legal prohibitions against homosexuality. Ms R’s case highlights the cultural and psychological conflicts arising from these societal pressures.

Psychodynamic Considerations: Ms R’s actions may have stemmed from a desire for attention and care amidst her challenging personal circumstances. The endocrinology team’s thorough investigation reflects the cultural emphasis on finding biological causes, but the eventual diagnosis strained the doctor–patient relationship, underscoring the complexities of treating such cases in resource-limited settings.

**Conclusion:** This case underscores the complex interplay of gender incongruence and factitious disorder within a restrictive socio-religious context. It highlights the importance of culturally sensitive, multidisciplinary care, the need for further research on gender identity in conservative settings, and deeper exploration of the psycho-dynamic relationships between patients and healthcare providers.

